# *In vitro* analysis of the influence of the thermocycling and the applied force on orthodontic clear aligners

**DOI:** 10.3389/fbioe.2023.1321495

**Published:** 2023-12-20

**Authors:** Patricia Cintora-López, Patricia Arrieta-Blanco, Andrea Martin-Vacas, Marta Macarena Paz-Cortés, Javier Gil, Juan Manuel Aragoneses

**Affiliations:** ^1^ Faculty of Dentistry, Alfonso X El Sabio University, Villanueva de la Cañada, Spain; ^2^ Faculty of Medicine and Health Sciences, Bionegineering Institute of Technology, International University of Catalunya, Barcelona, Spain

**Keywords:** polymers, orthodontic appliances, removable, clear aligner appliances, mechanical properties, creep

## Abstract

The mechanical properties of polyurethane dental aligners have been studied in an oral environment at 37°C and subjected to thermal cycling between 5°C and 55°C for long periods of time at different mechanical stresses. The aim is to determine the efficacy of the orthodontic aligner at different stress levels, the effect of thermal cycling with therapy time on tooth position correction. Sixty aligners with the same design were studied applying tensions of 0, 3 and 30 N and determining the deformation at different times from 1 to 760 h. Half of these aligners were subjected to stresses submerged in artificial saliva at 37°C and the other half were subjected to thermal cycles between 2°C and 55°C in salivary medium. Deformation was determined using a high-resolution stereo magnifier and ImageJ image analysis software. Water adsorption by the polyurethane was determined at the different test times. The results showed that in the unloaded aligners there is no appreciable deformation, but with thermal cycling there is a light shrinkage of the aligner due to the semi-crystallization process (ordering of polymeric chains) of the polyurethane. When applying loads of 3 and 30 N, creep curves with constant deformation transition zones can be seen. The transition zones decrease as the applied mechanical load increases. In addition, the significant effect of thermal cycling on the reduction of the transition zone of the aligners has been demonstrated. The transition zones are optimal for dental correction as constant stresses are exerted for tooth movement. The effect of thermal cycling shortens the constant deformation zone and reduces tooth alignment time. It was observed that the absorption of water in the aligner is constant after 1 h of immersion and does not exceed 0.4% by weight of absorbed water.

## 1 Introduction

Orthodontic systems of clear aligners and retainers combined with digital planning systems turn out to be the present and future of orthodontics. Effective orthodontic therapy necessitates a thorough understanding of the materials for our patients, without adequate knowledge of the materials used in our patients, their properties, and biocompatibility. Currently, the digital manufacturing method uses CAD-CAM (Computer Aided Design Computer Aided Manufacturing) technology and digital workflow protocol. The digital image is obtained either through an intraoral scanner or indirectly through buccal impressions in high-quality polyvinyl siloxane (PVS) material which are then digitally scanned to provide a digital model (.stl). Teeth movements will be planned with the digital dental model using digital CAD platforms and generating virtual sequential models with the teeth in the desired positions, planned for each stage. Printing 3D models of each virtual configuration through CAM technology involves incorporating subtractive (milling) or additive (3D printing) manufacturing techniques (Hartshorne and Wertheimer, 2022).

The performance of aligners depends on the composition of the material used for their manufacture, being directly dependent on the manufacturing process. The most widely used is the conventional vacuum thermoforming method that includes molding the thermoplastic material into physical models (Tartaglia et al., 2021). Thermoplastic Polyurethane (TPU), composed mainly of di- and tri-isocyanates and polyols, is another extremely versatile polymer with advantages such as excellent mechanical and elastomeric characteristics, chemical and abrasion resistance. TPU is the material that was one of the first to be used and has been evolving to this day. One of its advantages is that when subjected to a load, TPU changes shape but can recover, when the load is removed it elongates and recovers due to its flexibility. The material also features high tear and fracture strength (Bichu et al., 2022).

The changes suffered by orthodontic materials when they are introduced into an electrolytic medium such as the oral cavity, as well as the effect they suffer due to masticatory stress and other environmental changes, such as temperature, have been extensively studied by numerous authors in order to evaluate the effectiveness of the materials and the effect of the degradation of its components on the health of patients with orthodontic appliances. Most of these studies are carried out analyzing the effect on the components of fixed multibracketts appliances in different brands and materials (Pascual et al., 1999; Manero et al., 2003; Gil et al., 2004; Djeu et al., 2005; Keim et al., 2014; Abbate et al., 2015; Han, 2015; Li et al., 2015; Hennessy et al., 2016; Gu et al., 2017; Lanteri et al., 2018). With the increase in orthodontic treatment with aligners, it is necessary to enhance the studies in which the composition of its material is analyzed when it is introduced into the oral cavity and the degradation of its components based on various environmental stimuli to which they are subjected in the oral cavity (Eliades et al., 1999; Schuster et al., 2004; Eliades et al., 2007; Eliades et al., 2009; Gracco et al., 2009; Rossini et al., 2015; Halimi et al., 2016; Buxadera-Palomero et al., 2017; Eliades and Bisphenol, 2017; Yi et al., 2018; Francisco et al., 2022).

Invisaling^®^ system clear aligners are thermoplastic polymers based on polyurethanes, aromatic multilayer thermoplastic polyurethane, methylphenyl diisocyanate and 1,6-hexanediol plus additives (Raghavan et al., 2017). They are characterized by having higher values of hardness and elastic modulus and index, but lower values of creep resistance compared to other materials also used as aligners. Thermoplastics have excellent aesthetics and adaptability to different shapes that are widely used for orthodontic treatment. Since Kesling in 1945 produced the first aligner made of elastic polymer (Shalish et al., 2012), there has been an important development in materials and especially in CAD-CAM manufacturing processes, thermofabrication, 3D printing, etc. that have been fundamental to improve orthodontic treatments (Kesling, 1945; Elkholy et al., 2015; Gomez et al., 2015). In 2013, Invisalign^®^ began using SmartTrack as a material for its aligners, a multilayer polymer that provides light and constant force and superior control over tooth movement (Simon et al., 2014).

Although there are numerous studies on the biological behavior of clear aligners with different thermoplastic polymeric materials, there is no much evidence on their mechanical properties (Align Technology, 2023). Due to this lack of evidence, it is important to carry out research which analyze the behavior of clear aligners in the oral environment, and the influence of thermal cycles. The hypothesis of this research was that polyurethanes, being thermoplastic materials, would produce elongation in the aligner by exerting constant tension on its walls. Furthermore, the effect of temperature can influence the movement of polymeric chains, affecting the correction of dental position. The aim is to analyze in an artificial mouth the influence of the temperature and masticatory forces on the behavior of the clear aligner characteristics.

## 2 Materials and methods

The aim is to determine the creep of the aligner, i.e., the increase in deformation when applying a constant force for long periods of time. This mechanical behavior simulates the effect of stresses created on the aligner in the mouth over time and can be determined when the aligner is active. For this purpose, different forces will be applied by means of a spring calibrated with a constant force, determining the elongation increases at different times. The tests will be carried out at a constant temperature and thermocycled. In this way it will be possible to determine the effect of the variations of the temperatures on the mechanical creep behavior of the aligners (Morresi et al., 2014).

An *in vitro*, experimental transversal research was carried out. Due to the *in vitro* design of the study, the approval of the Ethics Committee was not necessary. According to the calculation of experiments, a total of 60 identical clear aligners were studied. These aligners were manufactured by Align Technologies, Inc. (Tempe, Arizona, USA) with polyurethane 1,6-hexanedial methylene diphenyl diisocyanate. The thickness of the clear aligners was 0.72 mm.

**FIGURE 1:**
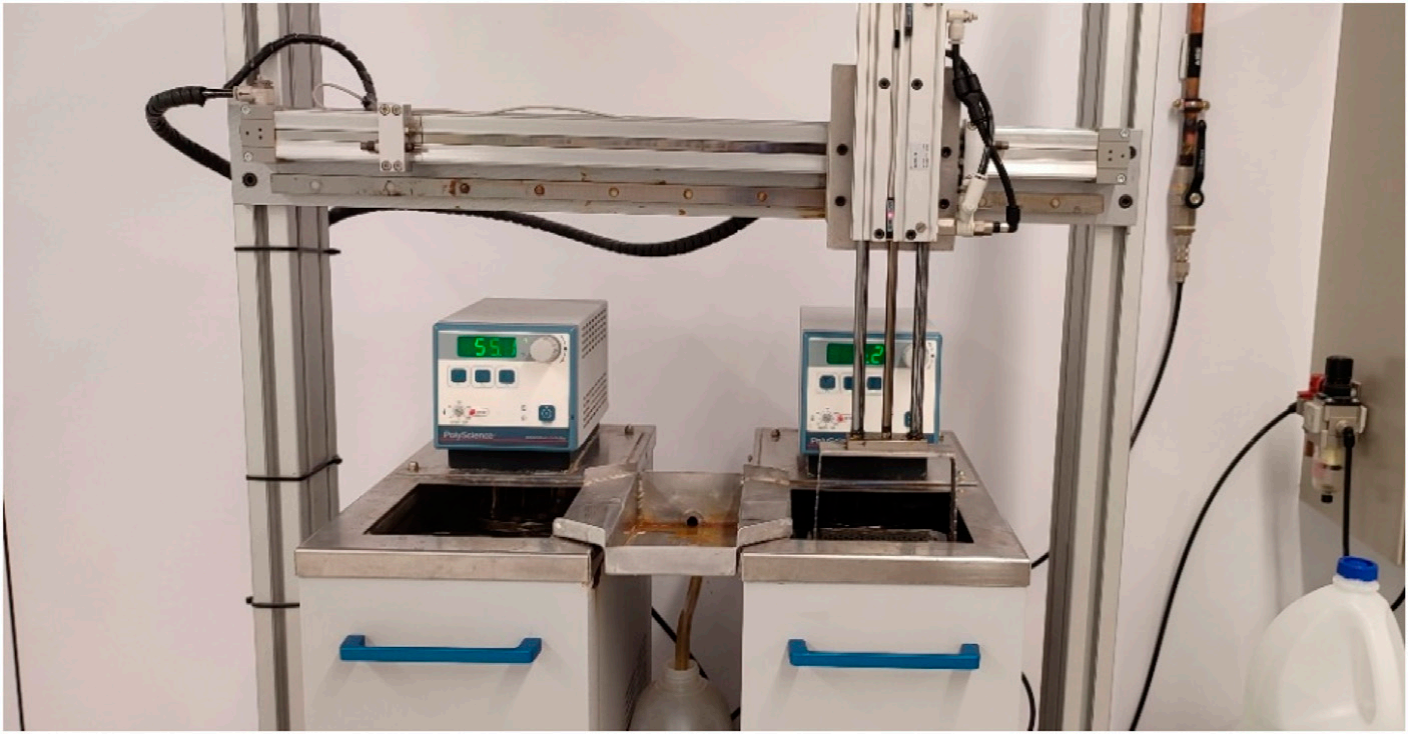
Thermocycling equipment. The thermocycles studied in artificial saliva were between 2°C and 55°C being at these temperatures for 30 s. At the different test times, the aligners were removed, and the elongation was measured.

Thirty samples were placed in an artificial saliva container at constant temperature of 37°C. The chemical composition of artificial saliva can be seen in Table 1 (Murakami et al., 1983). Thermal thermocycling was applied to 30 random samples for 600 cycles (1,200 immersions) considering in this case 20 temperature changes in the patient’s mouth for 30 days. For the thermocycling study, equipment manufactured in the dental laboratory of the International University of Catalunya (UIC, Sant Cugat del Vallés, Spain) was used to control the thermal cycles by immersion in a thermostatic bath with a controlled temperature at 2°C for 30 s and another 30 s in a bath at 55°C (Figure 1).

**TABLE 1:** Chemical composition of artificial saliva.

Chemical composition	g/dm^3^
K_2_HPO_4_	0.20
KCI	1.20
KSCN	0.33
Na_2_HPO_4_	0.26
NaCl	0.70
NaHCO_3_	1.50
Urea	1.50
Lactic acid	Until pH = 6.7

**FIGURE 2:**
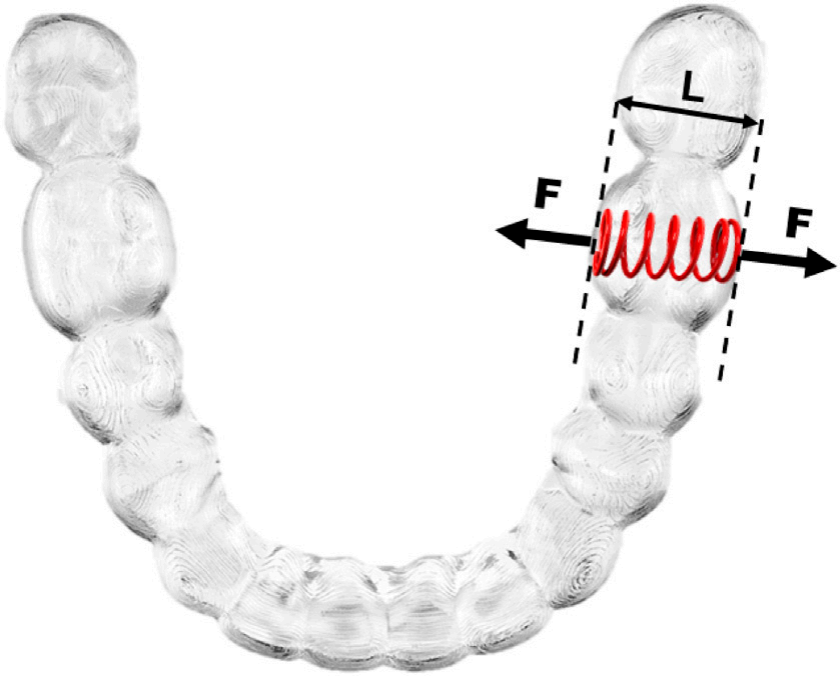
Diagram of the mechanical test performed on aligners (F: Forces; L: length).

Mechanical tests were carried out at 0 N, 3 N and 30 N applied to the first left molar (3.6). We studied 10 inserts for each were studied. The force is exerted with a calibrated spring that exerts tensile stress on the mold walls as can be seen in Figure 2. The springs are calibrated by a highly sensitive Adamel Lhomargy dynamometer (Adamel Lhomargy ST2507-E23, Saint Baldolph, France). These stresses simulate the stresses exerted by the tooth on the template. Elongations caused by applied force and/or thermal cycling were determined by observing the samples under a high-precision stereo magnification Q-star high-resolution system using ImageJ software for measurements (Bethesda Q-2022-ST18965, Maryland, USA). Two marks are made on the aligner with a diamond-tipped indenter at two points. The sample is positioned in such a way that there can be no movement and it is ensured that the electronic source is always at the same angle. ImageJ is used to determine the difference between points in the unloaded state, obtaining the distance between the points automatically, which corresponds to the initial distance.

ImageJ is the world’s fastest pure Java image processing program. It can filter a 2048 × 2048 image in 0.1 s. That’s 40 million pixels per second. 8-bit grayscale or indexed color, 16-bit unsigned integer, 32-bit floating-point and RGB color. Tools are provided for zooming (1:32 to 32:1) and scrolling images. All analysis and processing functions work at any magnification factor. Create rectangular, elliptical or irregular area selections. Create line and point selections. Edit selections and automatically create them using the wand tool. Draw, fill, clear, filter or measure selections. Save selections and transfer them to other images. Supports smoothing, sharpening, edge detection, median filtering and thresholding on both 8-bit grayscale and RGB color images. Interactively adjust brightness and contrast of 8, 16 and 32-bit images. Measure lengths in this case and standard deviation. Calibrate using density standards. Split a 32-bit color image into RGB or HSV components. Merge 8-bit components into a color image. Convert an RGB image to 8-bit indexed color. Apply pseudo-color palettes to grayscale images (Vallet-Regí et al., 2005).

The aligner is subjected to a constant force and the distance at different test times is determined. With the new distance the software calculates the elongation. The sensitivity of the distances calculated by the equipment and the software was 0.001 mm and has been used in different research studies that require high precision (Vallet-Regí et al., 2005; Godoy-Gallardo et al., 2016a; Herrero-Climent et al., 2023). Measurement times were: 0, 1, 2, 5, 10, 20, 24, 48, 72, 96, 120, 144, 168, 216, 240, 264, 268, 312, 336, 384, 432, 480, 528, 576, 624, 672, 720 h. The 720 h correspond to the hours of a month, the maximum application time. Elongation was calculated by determining the difference between initial separation between two points of the aligner and the distance between the same points after the experimental time has elapsed. The points are always the same and were identified by a small incision in the surrounding area (Herrero-Climent et al., 2023).

The water absorption of the clear aligners at different times without thermal cycling was studied using 20 mm × 20 mm samples. Samples were stored in a desiccator until constant weight (Initial weight = W1) was reached and then immersed in artificial saliva at 37°C for 1 min. After the different times they are dried with a cloth and weighed (Second weight = W2). They are weighed on a balance with a sensitivity of 0.00001 g (Sartorius x1000, Barcelona, Spain) and the water absorption (WA) is calculated according to the formula (Padrós et al., 2020):

$$\text{WA}=\frac{W_{2}-W1}{W1}x100$$

Data were statistically analyzed using Student's t-tests with a 99.5% significance level (*p* < 0.005) and asympotics significance and Turkey multiple comparison tests to assess any statistically significant differences between sample groups. All statistical analyzes were performed using MinitabTM software (Minitab version 13.0, Minitab Inc., USA).

## 3 Results

**FIGURE 3:**
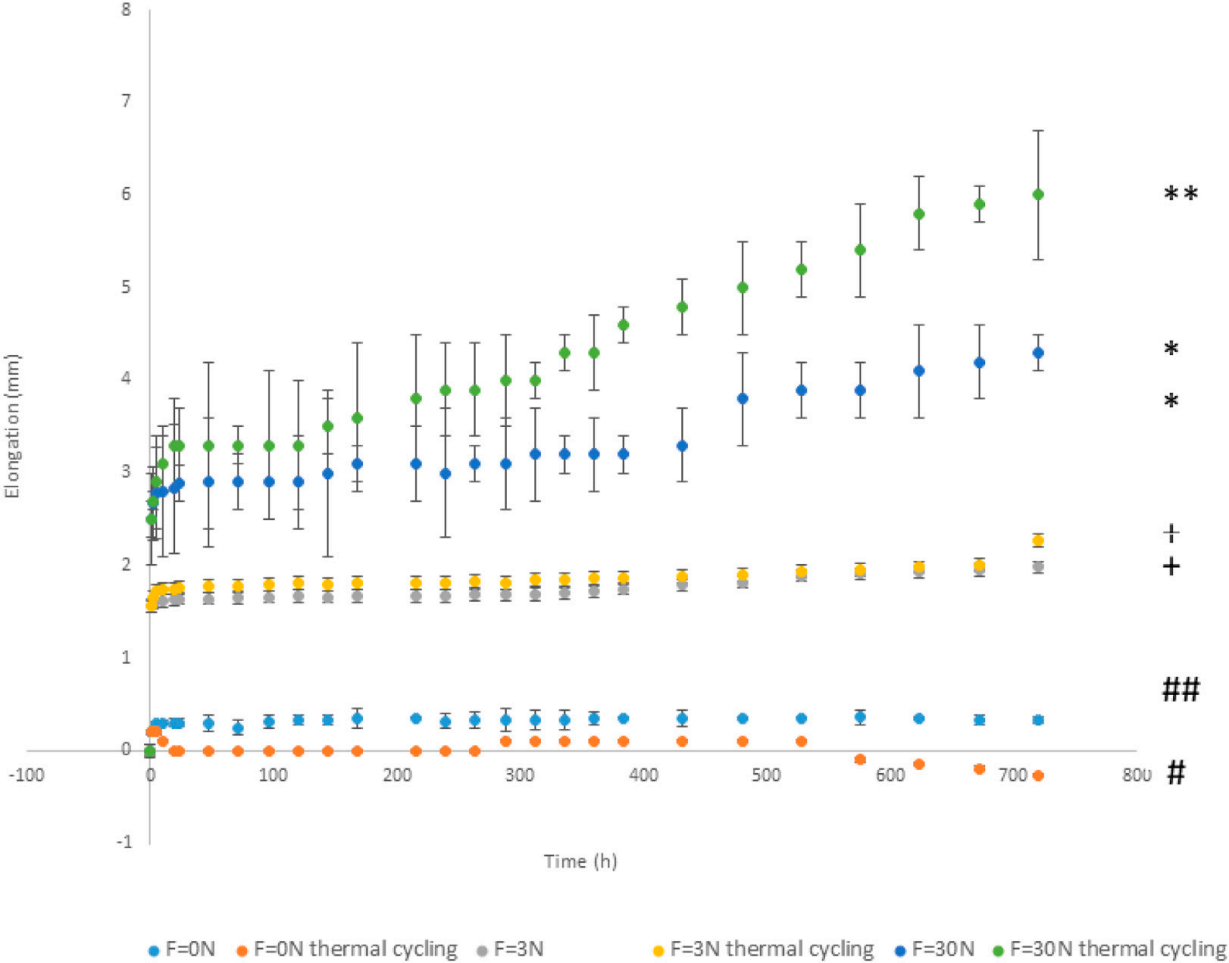
Elongation of clear aligner appliance without load and loaded at different forces without and with thermal cycling in relation to the time in hours. (Different signals indicate significance statistical differences with *p* < 0.05).

Figure 3 shows the elongation values with the test time for the aligner appliance unloaded and loaded at 3 and 30N with and without thermal cycling.

**FIGURE 4:**
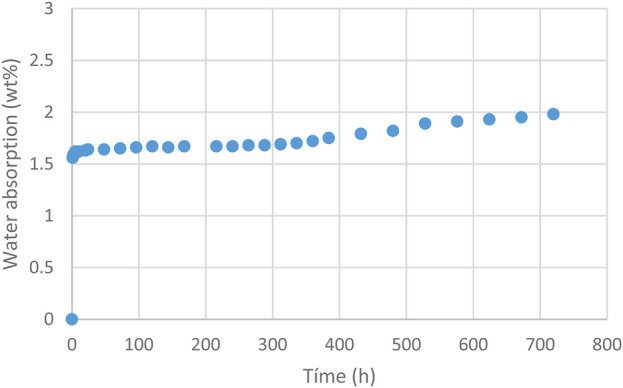
Water absorption up to 1 month.

The water absorption results of the artificial saliva give constant values from 5 h onwards, as can be seen in Figure 4.

## 4 Discussion

It can be observed that the samples that do not undergo loading and without thermal cycling, the elongations are practically negligible, and no changes in the shape of the template are observed. Samples without loading but which are thermally cycled show shrinkage of the template after 550 h of cycling. This shortening of the distances may be due to a partial chain folding process, called crystallisation, which decreases the distances.

The polyurethanes used are composed of two main polymeric segments: hard and soft chains. These two types of segments have different chain dynamics with repulsive interaction towards each other (Huang et al., 2012). The hard chains, with high extent of hydrogen bonding, have higher glass transition temperature (Tg) compared to soft chains and act as a physical cross-linker of soft segments. The repulsive interaction between hard and soft segments results in a decrease in crystallization of the soft segments (Lu et al., 2017). Salvekar et al. (Salvekar et al., 2017) determined for polyols composed of two different chains, their final physical and thermodynamic properties depend on the extent of phase separation, and their relative composition. To control the extent of phase separation for two different polyol chains, there are different procedures such as copolymerization with different structures, from random to block copolymers, and directed self-assembly of homopolymers with block copolymers of the same chemistry as homopolymers (Ge et al., 2012).

Some polyurethanes present shape memory property is due to the sensitivity of polyurethane to stimuli to retain an induced shape at a constant low temperature for a time: it also exhibits the ability to recover the original shape after removing the external stimulus force in the reheating process (Xie and Rousseau, 2009; Wu et al., 2017). These polymers are known for their ability to recover shape while maintaining constant stresses. In orthodontics, low and constant tensions in tempo are optimal to cause tooth movement; this same behavior is achieved with superelastic NiTi orthodontic wires (Gil and Planell, 1993; Gil et al., 2012; Gil et al., 2013; Bellini et al., 2016; Gil et al., 2018). The justification for the high shape resilience of polyetherimides is the presence of hard domains that act as physical cross-linkers to prevent chain slippage (Wu et al., 2017). The soft chains are responsible for fixing the temporary shape over a range of temperature (Tg) or crystallization temperature (Tc) (Ge et al., 2012; Wu et al., 2017). Thermal cycling below the Tg temperature will allow chain folding during heating, which reduces the dimensions of the aligner.

The materials with 3N mechanical loading show an initial deformation of the template due to the application of the load at time zero. Afterwards, a slight increase in elongation is observed with a very flat transition zone. This plateau is ideal for orthodontic treatment as the stress on the tooth does not cause an increase in elongation. At longer times, an increase in elongation is observed due to the creep of the polymeric material (Lombardo et al., 2012; Ihssen et al., 2019; Papadopoulou et al., 2019). This behavior is accentuated when thermal cycles are applied to the insole. At times longer than 600 h, a more intense increase in elongation is observed.

The insoles subjected to 30 N undergo a greater elongation than in the case of 3 N and a plateau suitable for orthodontic therapy can also be seen. After 500 h, a slight change in the slope of the deformation rate is observed. When subjected to this 30 N load and the material is thermally cycled, the plateau is reduced and from 450 h of treatment the elongation increases considerably. At high loads, as in this case, the thermal cycling has an intensifying effect on creep, which is why the treatment times under these conditions have to be shorter.

As can be seen, the aligners follow the typical creep graph, which has three stages (Espinar-Escalona et al., 2013; Iijima et al., 2015; Lombardo et al., 2015; Dalaie et al., 2021).

The first stage corresponds to a sudden increase in deformation when mechanical load is applied.

The second stage is the plateau zone which is the zone where the mechanical force exerted on the teeth is practically constant. It is called the transition phase and is the most suitable force for the correction of tooth positions. Constant, low intensity forces are the most suitable for tissue accommodation and the correct apposition and deposition of the bone that causes orthodontic movements.

The third stage is a stage of gross growth where the aligner may have lost thickness due to constant tension, which has produced a rearrangement of the polymeric chains causing a sudden increase in deformation. This stage is ineffective for any tooth movement and the aligner has ceased to perform its function.

This creep treatment occurs because polyurethane is a thermoplastic polymer, i.e., the temperature causes the polymer chains to align and can modify their shape and therefore the stresses exerted on the teeth or molars. These movements of polymer chains are produced by the effect of temperature. When the polyurethane is not mechanically loaded, the effect of temperature is the folding of chains in the so-called pseudocrystalline zones. This chain folding causes a decrease in volume, and it is for this reason that the aligner at 0N with thermal cycling up to 55°C shrinks (Lombardo et al., 2017; Dalaie et al., 2021).

The chain folding process does not occur when the polyurethane is subjected to mechanical stress as it prevents folding due to the directionality of the force exerted. For samples subjected to mechanical stress, thermal cycling facilitates the movement of the polymer chains in the direction of the stress, causing a faster creep of the aligner and reducing the plateau of the aligners, thus impairing tooth movement (Hodge et al., 1996; Godoy-Gallardo et al., 2016b; Jaggy et al., 2020).

For a given load and level of deformation, a material can be considered as symmetrical if the creep strain does not vary over time while an asymmetrical material presents a change in creep strain rate. Previous studies reported the asymmetrical behavior of polyurethane (Kasgoz, 2021), which is related, to the general chemical nature of the polymer, for example, including interactions among the polymer chains over time, chemical bonds and others. At 25°C, this grade of polyurethane displayed an asymmetric nature at only 30 N of load, which is an extreme condition, not commonly achieved when in service. In this case, an increase in the elongation rate can be observed during the test run.

The tests were performed at thermal cycles to simulate an accelerated aged condition for this grade of polyurethane and were stipulated according to previous studies, where better ageing responses were obtained at this level of temperature (55°C) (Jia et al., 2011). As seen on Figure 3, under 30 N and thermal cycling accelerated ageing conditions all specimens tend to present an increase of elongation. Creep strain rate results in both tested temperatures did not show a creep fracture point, which demonstrate that in the aforementioned conditions, the material used did not fail even after the lengthy duration of the test. However, the aligner losses the activation to move the teeth.

The modeling of the creep behavior of polyurethanes must take into consideration two contributions: the viscoelastic behavior and the long-term behavior, i.e., the influence of time. The modeling of viscoelasticity integrates the structure-properties interactions, the share of elastic and viscous deformation in the total deformation and the relaxation coefficient. Many viscoelastic models have been developed and used by various authors in order to define the relationship between viscoelastic structure and polyurethane characteristics (Wu et al., 1994; Findley and Davis, 2013).

The best fitting model is the four-element Burger model (Wu et al., 1994; Jhao et al., 2022; Cui and Clyne, 2023), which is a simple combination of the Maxwell and Kelvin-Voigt models.

Under the condition of linear deformation, the total strain of a viscoelastic solid may be given as the sum of immediate elastic deformation of Maxwell spring (ε_M1_), delayed elastic deformation of Maxwell dashpot (ε_M2_) and viscous deformation of Kelvin unit (ε_K_) (Eq. 1).

$$\varepsilon \left(t\right)=\varepsilon_{M1+}\varepsilon_{K+}\varepsilon_{M2\;}$$ (1)

Considering the combination of the Maxwell and Kelvin-Voight elements in the Burger model, the mathematical representation of the Burger model may be given as follows;

$$\varepsilon \;\left(t\right)=\frac{\sigma }{E_{M}}+\frac{\sigma }{E_{K}}\;\left[1-\exp\frac{-E_{K}t}{\eta_{K}}\right]\frac{\sigma t}{\eta_{M}}$$ (2)

where E_M_ and η_M_ are the modulus and viscosity of the Maxwell spring and dashpot, respectively; E_K_ and η_K_ are the modulus and viscosity of the Kelvin spring and dashpot, respectively, and σ is the applied stress value.

From the values in Figure 4, it can be confirmed that water absorption by the polyurethane is constant from the first hours of placement, reaching values that do not exceed 0.4% wt of the polyurethane composition. Therefore, the mechanical behavior studied is not affected by water absorption but depends fundamentally on the composition of the polymer, the stresses applied and the thermal cycling of the aligner (Mima et al., 1983; Skaik et al., 2019; Cui and Clyne, 2023).

Several studies have been carried out analyzing different materials of invisible aligners for orthodontics, observing creep phenomena for all of them. Cianci C. et al. (Cianci et al., 2020) studied the compression behavior under cyclic loading of Polyethylene terephthalate-glycol (PET-G) -one of the most famous and used thermoplastic- and used as invisible aligners. It was observed for the first day of testing of the aligners that stiffening effects occur while cyclic loading progresses. It was also observed that stiffening decreases during the non-loading time between two successive sessions of test while stiffening effects are observed again when a new set of cycles is applied to the aligners. One of the results obtained was that in the case of testing with saliva saliva an higher stress recovery is observed between two subsequent loading sessions; moreover, the hygrothermal environment showed a contribution to reduce the stress accumulation effect during the test. In our case we have observed that the influence of the thermal cycles accelerates the creep processes reducing the transition straight line which is the desired one to provoke the activation of tooth alignment.

Other investigations (Lombardo et al., 2017; Albertini et al., 2022) carried out for different aligner materials show behaviors like those observed in our results, although the tests have been carried out in short periods of time (24 h) in all the polymers released a significant amount of stress during the 24-h period. Stress release was greater during the first 8 h, reaching a plateau that generally remained constant. The single-layer materials, F22 Aligner polyurethane (Sweden & Martina, Due Carrare, Padova, Italy) and Duran polyethylene terephthalate glycol-modified (SCHEU, Iserlohn, Germany), exhibited the greatest values for both absolute stress and stress decay speed. The double-layer materials, Erkoloc-Pro (Erkodent, Pfalzgrafenweiler, Germany) and Durasoft (SCHEU), exhibited very constant stress release, but at absolute values up to four times lower than the single-layer samples tested. Albertini et al. (Albertini et al., 2022) conducted studies at longer periods of time at constant temperature determining that all the materials that we tested showed a rapidity of stress decay during the first few hours of application, before reaching a plateau phase. The polyurethane showed the greatest level of final stress, with relatively constant stress release during the entire 15-day period. Further research after *in vivo* aging is necessary in order to study the real aligners’ behavior during orthodontic treatment. These results show similar behaviors to those obtained without thermal cycling and as they suggest could be influenced by thermal cycling in salivary medium as in this work, we have been able to complete the research of Albertini et a (Albertini et al., 2022).

It has been possible to observe the consequences of the structural changes of semi-crystallization with the volume contractions of the aligners when the material is not subjected to mechanical loading. These results could be compared with the work of Condo et al. (CondoPazzini et al., 2018). These authors characterized the samples by means of Fourier transform infrared spectroscopy, micro-Raman spectroscopy, X-ray diffraction, tensile and indentation strength test. The results showed an increase of crystalline fraction producing an increase of hardness and hyper-plasticity.

This research work has several limitations as we have tried to simulate the oral environment in which the aligners work. However, chewing stress levels have not been considered, as well as the bacterial environment to which the aligner may be subjected. One of the limitations of this study is the determination of dental forces and occlusal forces. A good model is the study by Duanmu Zheng et al. (Duanmu et al., 2021) where they developed a biomechanical model for the analysis of occlusal stresses. Future work could use this model to develop the behavior of aligners under the conditions described in this contribution. In any case, the mechanical behavior of the aligners has been verified and the hypothesis put forward in the study has been confirmed.

## 5 Conclusion

Polyurethane dental aligners have characteristic creep curves with transition zones for loads of 3 and 30 N. These plateaus, which are optimal for dental alignment, are reduced by the effect of the load and thermal cycling. It was found that the water absorption in the inserts is constant after 1 h and reaches values of 0.4% by weight of the polyurethane. It has also been confirmed that thermal cycling in the insoles without mechanical stress is susceptible to shrinkage due to semi-crystallisation processes of the polymeric material.

## Data Availability

The raw data supporting the conclusion of this article will be made available by the authors, without undue reservation.
